# Supplementary material for: Prediction and stratification of longitudinal risk for chronic obstructive pulmonary disease across smoking behaviors
Source: Nat Commun. 2023 Dec 14;14:8297. doi: 10.1038/s41467-023-44047-8 (PMC10721891; doi:10.1038/s41467-023-44047-8)
Supplement: Supplementary file 3 — Description of Additional Supplementary Files [file 41467_2023_44047_MOESM3_ESM.pdf]

Description of Additional Supplementary Files:

Supplementary Data 1: Summary statistics from exposure-wide association study.

Supplementary Data 2: SERS model weights, responses in bold are significant ($p<0.05$) in the multivariable model.

Supplementary Data 3: Hazard ratio of each SERS decile compared to the first decile in the EUR evaluation subset.

Supplementary Data 4: Hazard ratio of each PGS decile compared to the first decile in the EUR evaluation subset.

Supplementary Data 5: Prediction of COPD incidence in subsampled populations of the UK Biobank (N=1500). The absolute C index and its standard errors (SE) are shown in each model without adjusting for covariates.
